# A campus digital twin framework for visualizing and managing anxiety-related stress among college students

**DOI:** 10.3389/frai.2026.1796722

**Published:** 2026-08-19

**Authors:** Yuchen Wang, Xinyue Ye

**Affiliations:** 1 Department of Landscape Architecture and Urban Planning & Center for Geospatial Sciences, Applications and Technology, Texas A&M University, College Station, TX, United States; 2 Department of Geography and the Environment & Alabama Geospatial AI & Information Science Hub, The University of Alabama, Tuscaloosa, AL, United States

**Keywords:** anxiety-related stress, campus digital twin, college students, geospatial analysis, public health

## Abstract

**Introduction:**

Anxiety-related stress among college students has become a significant public health concern, affecting student well-being, academic performance, and campus operations. However, the application of digital twins for campus mental health management remains limited. This study proposes a campus-scale digital twin framework to support monitoring, analysis, and management of anxiety-related stress among college students from a geospatial perspective.

**Methods:**

The framework is composed of three integrated layers. The data acquisition and management layer integrates aggregated stress-related indicators derived from wearable devices, campus mental health service records, and campus geospatial data. The modeling and simulation layer represents spatiotemporal dynamics of student stress patterns and enables scenario-based analysis. The user application layer provides interactive visualization and decision support tools for campus administrators, mental health professionals, and students.

**Results:**

Based on the proposed framework, we developed a digital twin visualization system with four primary functions. These include continuous monitoring of anxiety-related stress trends, campus-scale stress heatmap visualization to support spatiotemporal assessment and targeted intervention, digital twin-supported management and spatial planning of campus mental health facilities, and personalized low-stress path navigation with restorative space recommendations.

**Discussion:**

As a proof-of-concept, the prototype illustrates the potential feasibility of applying campus digital twin technologies to aggregated privacy-preserving stress visualization and decision support, and highlights the value of integrating geospatial intelligence with digital twin technologies. This study contributes a scalable and transferable framework that supports preventive mental health strategies, informed decision-making, and sustainable, healthy campus environments. Further empirical validation is required to evaluate system accuracy, usability, effectiveness, and ethical acceptability.

## 1 Introduction

In recent years, increasing academic demands and external influences such as the pandemic have contributed to a sustained rise in anxiety-related stress among college students, making it one of the most critical concerns for colleges and universities (Tan et al., 2023). As a result, identifying effective strategies to mitigate and intervene in student anxiety and stress has become an important research topic in public health. In addition to anxiety driven by traditional factors such as academic workload and financial pressure, existing research indicates that anxiety and stress levels among college students are also significantly associated with characteristics of the campus-built environment and their daily spatial experiences (Liu et al., 2022).

However, existing research has predominantly investigated anxiety and stress among college students through individual-level health and behavioral indicators, with limited attention to shared spatial contexts or environmental influences (Haidar et al., 2018). In addition, restorative environment theories such as Attention Restoration Theory (ART) and Stress Reduction Theory (SRT) demonstrate that environmental factors, including natural settings and green space, can help stress reduction and attention restoration (Roe and Aspinall, 2011). Thus, the lack of attention on environmental factors constrains the ability to develop spatially informed interventions and to advance sustainable and healthy campus environments. In parallel, rapid advances in digital technologies have led to the maturation of sensors and devices capable of monitoring individual emotional stress and changes in the spatial environment. These developments create new opportunities for digital twin technologies to support the monitoring, analysis, and management of emotional wellbeing among college students.

A digital twin (DT) is a real-time virtual representation of a physical process or system (Batty, 2024; Goodchild et al., 2024). Since Michael Grieves formally introduced the concept of digital twin for Product Lifecycle Management (PLM) at the University of Michigan in 2002 (Grieves and Vickers, 2016), DT has been widely applied in fields such as manufacturing (Cimino et al., 2019; Kritzinger et al., 2018), healthcare (De Benedictis et al., 2023; Sun et al., 2023), transportation (Feng et al., 2023; Irfan et al., 2024) and urban planning (Schrotter and Hürzeler, 2020; Ye et al., 2024). More recently, university campuses have emerged as an important application context for digital twin technologies, reflecting a growing research and practice interest.

Existing studies have primarily applied digital twins to specific campus operational subsystems, including curriculum management (Wu et al., 2025), energy and waste management systems (Teke et al., 2023), sustainable comfort monitoring (Zaballos et al., 2020), water distribution systems, (Singh et al., 2024) and campus heat stress monitoring (Gong et al., 2025). Also, from the perspective of mental health and stress management, although several studies have applied DT technology to stress monitoring and personalized management at the city level (Tan and Wei, 2025; Vallu et al., 2025), DT has not yet been systematically applied to address anxiety and stress among college students or to support mental health-related public health interventions at the campus scale. This absence highlights a critical research gap in the application of digital twin technologies to campus mental health management.

To address this gap, and in contrast to digital twin applications that emphasize individual-level diagnosis and treatment, this study develops a campus digital twin prototype to visualize and manage anxiety-related stress among college students from a geospatial perspective. This study makes contributions along three dimensions. First, we propose a proof-of-concept digital twin framework comprising three layers and demonstrate the methodology for operating the framework. Second, the prototype presents an interactive visualization dashboard and introduces four key functions: stress monitoring, spatiotemporal analysis, mental health facility management, and low-stress path navigation. Third, we use synthetic data to illustrate the potential feasibility of this framework, leaving real-world validation for future work. Overall, the proposed digital twin prototype aims to support the monitoring of student mental stress conditions, enhance decision-making for campus mental health administrators, and inform targeted public health intervention strategies. Through this approach, the study seeks to contribute to the development of a healthier, more sustainable, and data-informed campus mental health environment.

## 2 Methodology

This study introduced a proof-of-concept prototype for visualizing and managing anxiety-related stress among college students using campus digital twin technologies. A conceptual framework is proposed to illustrate the potential feasibility of this approach at the design stage. Given the privacy and complexity of multi-source health data integration, empirical validation using real-world data and user evaluation will be addressed in future work. The specific methodology is organized as follows:

### 2.1 Digital twin framework

**Figure 1:**
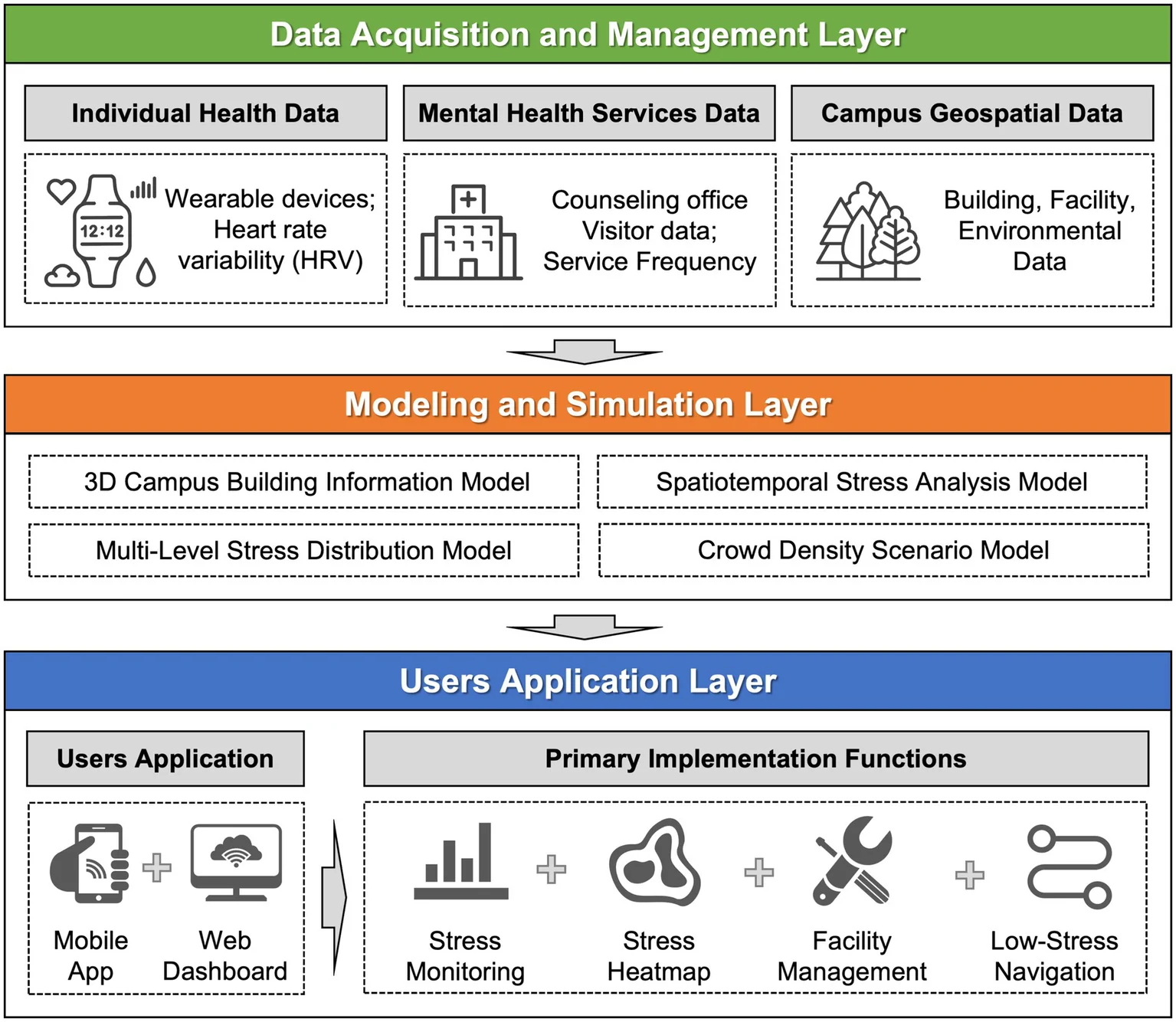
Campus digital twin framework for visualizing and managing anxiety-related stress among college students.

To visualize and manage anxiety-related stress among college students, the proposed campus digital twin framework is designed as a three-layer architecture (Figure 1), including data acquisition and management layer, modeling and simulation layer and users application layer.

- *Data Acquisition and Management Layer*. This layer focuses on collecting and processing mental health-related data from college students, including physiology information from wearable devices such as Apple Watches that capture the heart rate variability (HRV). It also integrates campus counseling visitation and service frequency records. In parallel, geospatial data on campus buildings, facilities, and environmental features are gathered to support development of a 3D campus model. Moreover, the multi-source data are not fused into a single composite measure but serve distinct functional modules. For example, individual health data are used for stress monitoring, mental health services data for facility management, and campus geospatial data for 3D space modeling and path navigation.
- *Modeling and Simulation Layer*. Based on the collected and preprocessed data, a 3D campus building information model and a multi-level (individual-to-population) anxiety-related stress distribution model are generated. A spatiotemporal stress analysis model and a crowd density scenario model are then applied to analyze student stress scenarios during both regular academic periods and examination weeks.
- *Users Application Layer*. With college students, faculty, administrators, and psychologists as primary user groups, mobile applications and web-based dashboards support collaborative interaction. The dashboard provides multiple functions, including stress monitoring, stress heatmap visualization, facility management, and campus navigation services.

### 2.2 Anxiety-related stress measurement and modeling

Given the diversity of stress, we define anxiety-related stress as chronic physiological stress within this proposed framework. To better support campus public mental health management, this prototype focuses primarily on accumulated autonomic stress over extended periods, such as weekly, monthly, or examination periods, at both the individual and population levels. This system is not intended to capture acute, event-driven stress or clinically diagnosed anxiety disorders. Psychological emergency incidents are handled separately as operational records for administrative response rather than as inputs to the stress metric. In addition, heart rate variability (HRV) is selected as the primary physiological signal for anxiety-related stress, as it can be readily collected by wearable devices such as the Apple Watch and Google Fitbit.

Heart rate variability (HRV) is currently recognized by the academic community as an essential physiological indicator associated with anxiety disorders and mental health (Robinson et al., 2023; Tomasi et al., 2024a; Tomasi et al., 2024b). HRV represents the variation in the time intervals between consecutive heartbeats and reflects the balance between sympathetic nervous system (SNS) and parasympathetic nervous system (PNS) control of the cardiac sinoatrial node (Berntson et al., 1997). Related research reveals that lower HRV is associated with anxiety disorders and decreased well-being, whereas higher HRV reflects a relaxed mental status (Kemp and Quintana, 2013). Building upon this background, we propose the anxiety-related stress index for individual *i* at time *t*, as shown in Equation 1.

$$S_{i}(t)=1-\mathrm{HR}V_{i}(t)$$ (1)

where *HRV_i_(t)* is the min–max normalized heart rate variability of individual *i* at time *t*, scaled to [0,1]. The lower the *HRV_i_(t)* value, the higher the stress level *S_i_(t)* of individual *i*. Moreover, based on the stress metric *S_i_(t)*, we calculate average population-level stress indicators by aggregating individuals within a given campus area, thereby enabling population-level stress monitoring.

For the modeling and simulation layer, we further conduct spatiotemporal analysis and modeling across space and time. Using kernel density estimation, we map individual stress values *S_i_(t)* and their corresponding spatial locations to create the campus stress heatmap. Additionally, rather than adopting agent-based modeling, this prototype proposes a rule- and scenario-based approach. Under different temporal scenarios, such as examination or regular weeks and daytime or nighttime, we calculate the average stress metric for different campus zones. For example, the examination-week scenario is generated by raising the baseline stress levels of campus zones above their regular-week values. In addition, as course schedules are a key factor in student gathering (Wu et al., 2025), we assigned crowd density based on building types such as libraries and teaching buildings, as well as student class-schedule patterns. It should be noted that at the design stage, this prototype captures temporal patterns of chronic stress, such as weekly and monthly patterns, rather than performing real-time tracking of individual stress and movement. A fully dynamic simulation using real-time data will be the focus of future work.

### 2.3 Low-stress path navigation model

The low-stress path navigation model is grounded in restorative environment theories such as Attention Restoration Theory (ART; Kaplan and Kaplan, 1989; Kaplan, 1995) and Stress Reduction Theory (SRT; Ulrich, 1983; Ulrich et al., 1991). These theories provide evidence that low-stress routes with environmental factors such as greater natural elements, visual openness, and green space can support psychological restoration, stress reduction, and attention recovery, which provides students with opportunities for temporary mental disengagement from stressful academic settings. In addition, the idea of an optimal route navigation system has been widely used across various domains, including a health-optimal route recommendation system for PM2.5 exposure (Mahajan et al., 2019), an exposure-optimized route planning system that accounts for environmental exposures such as noise levels, air quality, and street-level greenery (Helle et al., 2023), and a thermal comfort-optimized campus walking route recommendation system based on Dijkstra and A* algorithms (Gong et al., 2025).

Building on these studies, and from the perspective of anxiety-related stress management, this prototype proposes a weighted least-cost formulation for a low-stress walking-path navigation system, which has been built in the digital twin platform. Specifically, we first build a graph *G = (V, E)* based on the campus road network, in which nodes represent intersections and edges represent walkable road segments. For each edge e, we select four cost indicators: walking distance *d(e)*, noise level *n(e),* crowd density *p(e)*, and greenness *g(e)*. All four indicators are min–max normalized to the range [0,1]. According to restorative environment theory, high noise *n(e)* and crowd density *p(e)* exacerbate stress, whereas greater greenness *g(e)* supports restoration and reduces stress. Finally, the optimal low-stress walking path P* is the path that minimizes the sum of edge costs. The details are shown in Equations 2, 3.

C(e)=d(e)+n(e)+p(e)+(1−g(e)) (2)

$$P^{*}=\arg\min_{P}\sum_{e\in P}C(e)$$ (3)

where the term *(1 − g(e))* ensures that each edge cost remains non-negative. Dijkstra’s shortest-path algorithm is employed to obtain the optimal low-stress walking path P*. It should be noted that these four indicators are weighted equally as an initial default in this prototype, and each weight can be adjusted for different scenarios in future work.

## 3 Prototype demonstration

**Figure 2:**
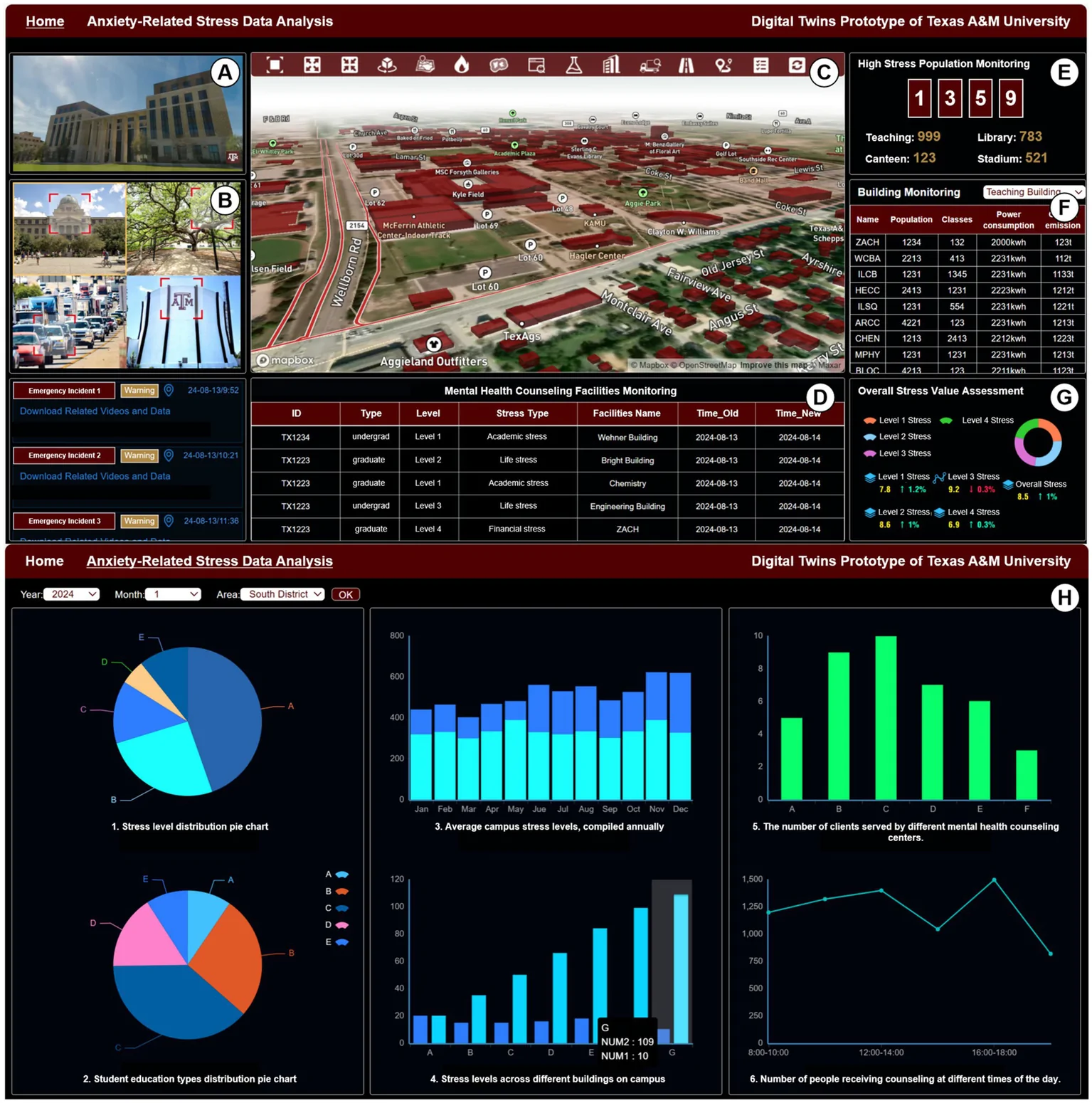
Campus anxiety-related stress visualization system: **(A)** Project showcase view; **(B)** Psychological emergency incident monitoring view; **(C)** Map interactive view; **(D)** Mental health counseling facilities monitoring view; **(E)** High stress population monitoring view; **(F)** Crowd stress monitoring in buildings view; **(G)** Overall stress value assessment view; **(H)** Anxiety-related stress data analysis view.

Based on the proposed digital twin framework and methodology, we design and build a prototype digital twin visualization system for anxiety-related stress among college students, developed using Texas A&M University as the deployment context (Figure 2). The system home page includes seven functional views, each with distinct functions. It should be noted that we use synthetic data rather than real-world health data to illustrate the potential feasibility of this prototype for demonstration at the design stage.

Among these views, Figure 2A presents the project showcase view. Figure 2B shows the psychological emergency incident monitoring view, supporting monitoring of anxiety and psychology-related emergencies on campus. Figure 2C displays the map interactive view, providing map interaction, information querying, geographic analysis, route planning, and navigation. Figure 2D presents the mental health counseling facilities monitoring view, reporting visitation counts and related information for each counseling facility. Figure 2E shows the high stress population monitoring view, which visualizes high-stress populations across campus locations, such as teaching buildings and libraries. Figure 2F presents the crowd stress monitoring in buildings view, displaying average crowd stress levels and corresponding energy consumption across buildings. Figure 2G illustrates the overall stress value assessment view, offering a comprehensive evaluation of stress types across the campus. Figure 2H presents the anxiety-related stress data analysis view, which integrates multiple data sources to analyze anxiety-related stress by type, time period, and region. Based on this dashboard, four primary functions are achieved, as described below.

### 3.1 Function 1: monitoring anxiety-related stress trends among college students

This function supports campus administrators, mental health service providers, and campus infrastructure staff through a dashboard-based design for monitoring anxiety-related stress trends among college students and informing decision-making. To protect student privacy, all visualizations and analyses rely on aggregated and anonymized data rather than identifiable individual records. For example, the interactive map view in Figure 2C presents the spatial distribution of overall stress levels across the campus, while Figure 2B highlights locations associated with high stress emergencies and mental health-related incidents, enabling timely administrative response without revealing identities. Figure 2E summarizes high-stress populations across building types, Figure 2G displays overall campus stress levels, and Figure 2H provides integrated analysis of anxiety-related stress patterns. Users can examine aggregated stress distributions, variations across student education groups such as undergraduate and graduate populations, spatial differences across campus zones, temporal patterns across months and days, and changes in counseling center visitation volume. By emphasizing population-level patterns rather than individual tracking, this function balances decision support with student privacy. Collectively, these features provide administrators with actionable and ethically responsible insights to support management and mitigation of anxiety-related stress.

### 3.2 Function 2: campus stress heat map and spatiotemporal monitoring and intervention

**Figure 3:**
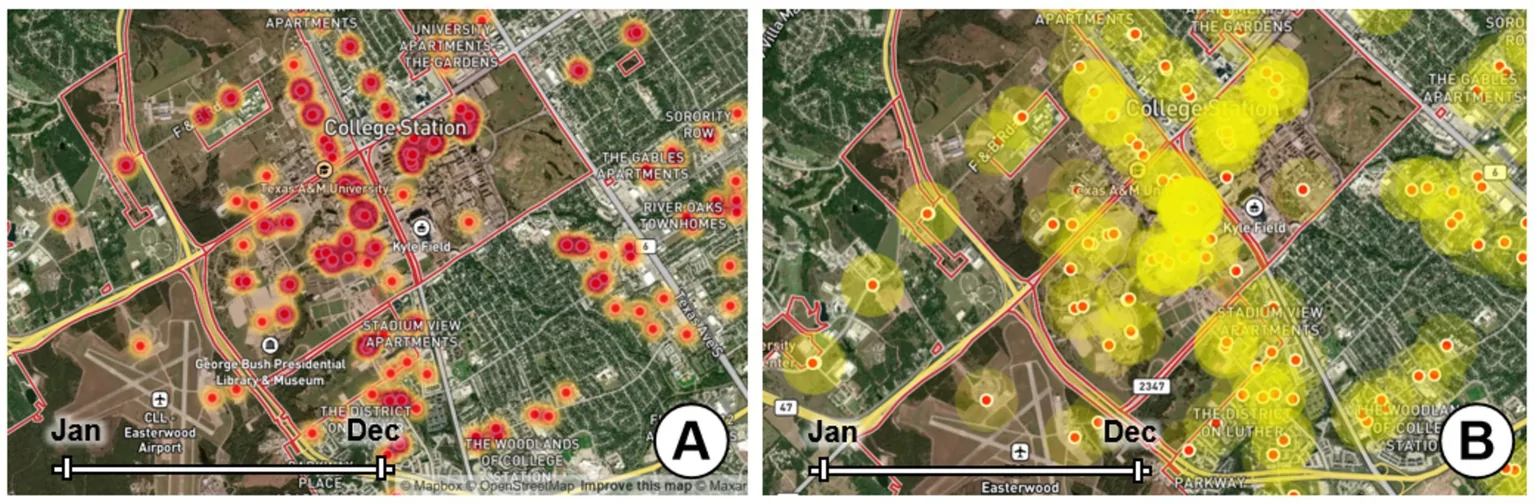
Visualization of anxiety-related stress distribution on campus: **(A)** Spatial distribution and heat map of high-stress college students; **(B)** Schematic diagram of spatial coverage for mental health facilities.

This feature is integrated into the map interaction view shown in Figure 2C and displays an aggregated heatmap of anxiety-related stress across the campus, enabling visualization of areas with relatively high and low stress levels without exposing individual-level information. The heatmap in Figure 3A can be overlaid with real-time contextual data such as traffic flow, pedestrian movement, and noise levels, together with spatial data from multiple sources including green spaces and teaching areas. In addition, the spatial coverage of mental health facilities shown in Figure 3B allows campus administrators to examine campus-wide stress patterns and to evaluate whether additional counseling or support services are needed in high-stress locations such as academic buildings, or whether temporary interventions should be implemented. A time slider located in the lower left corner of the interface enables users to view campus-wide stress distribution maps under different temporal conditions, including monthly variation from January to December, regular days and examination weeks, daytime and nighttime periods, and weekdays and weekends. By focusing on aggregated spatial and temporal patterns rather than individual trajectories, these functions support evidence-based decision-making while preserving student privacy.

### 3.3 Function 3: campus mental health facility management and spatial layout planning

**Figure 4:**
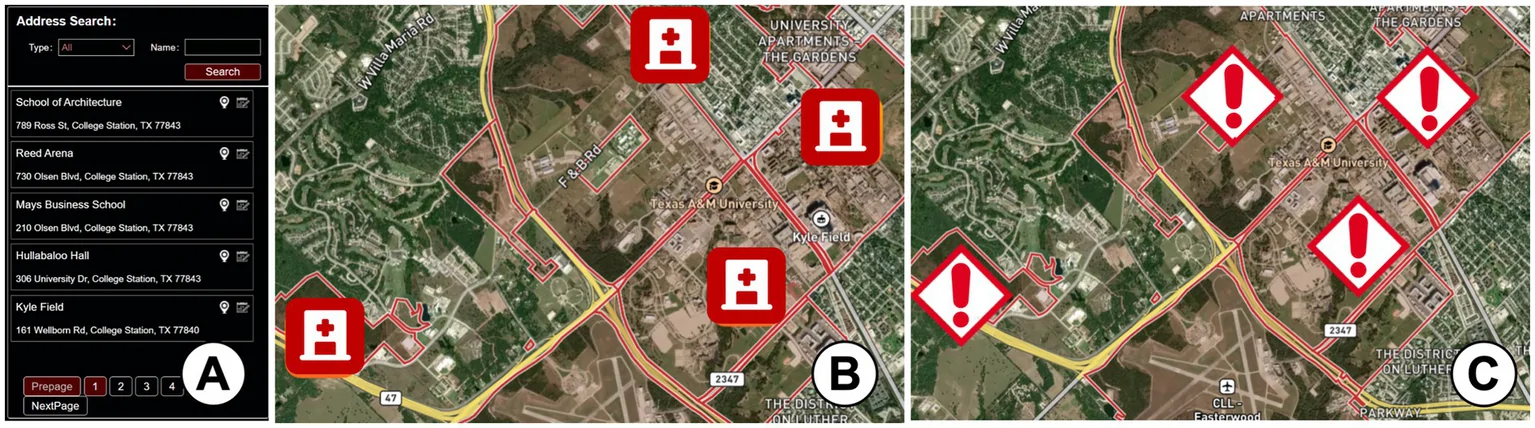
Facility management and spatial layout planning **(A)** Facility information retrieval view **(B)** Spatial distribution of major mental health facilities on campus **(C)** Spatial distribution map of mental health facility malfunctions and maintenance incidents on campus.

This function focuses on a dashboard-based design to support the planning and management of campus mental health facilities while protecting student privacy. Figure 2D presents aggregated real-time information on visitor volumes at mental health counseling centers and psychological clinics across the campus, together with summary attributes such as student academic levels, categories of anxiety-related concerns, and visit time periods. All information is presented at an aggregate level without revealing any individually identifiable records. Campus administrators can use these summaries to assess facility capacity and congestion, examine whether peak service demand aligns with periods of elevated campus-wide stress, and consider the deployment of new or temporary mental health facilities across different stress zones. Figure 4 is integrated into the interactive map view shown in Figure 2C. Figure 4A provides an information-retrieval interface for campus facilities, including counseling centers, campus hospitals, medical service points, sports and fitness facilities, and parks and green spaces. Figure 4B displays the spatial distribution of these facilities across the campus. To support infrastructure operations, Figure 4C presents an aggregated map of mental health facility malfunctions and maintenance incidents, enabling campus construction and maintenance staff to respond efficiently while ensuring that all facilities remain operational.

### 3.4 Function 4: low-stress path navigation and restorative space recommendations

**Figure 5:**
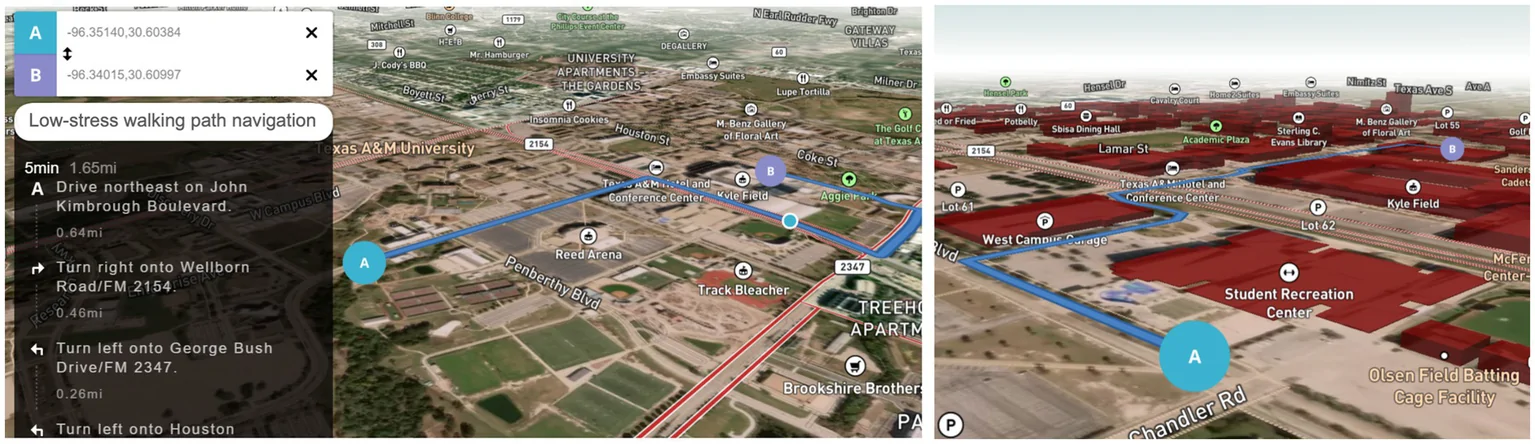
Low-stress path navigation and restorative space suggestion.

The fourth function supports college students in stress relief and navigation through low-stress routes using an interactive map view while preserving personal privacy. Figure 5 illustrates an example in which a student inputs a starting point and destination, and the system recommends an optimal low-stress walking route characterized by lower noise levels, greater availability of green spaces, and reduced crowd density, which can help alleviate emotional stress. Route generation is based on multidimensional sensor data describing sound levels, environmental conditions, and traffic characteristics across campus road segments. These data are processed collectively to avoid tracking individual movement patterns. Based on the routing algorithm, the system provides low-stress route options that balance walking distance and environmental quality, which is particularly useful during examination periods and class transitions. In addition, the system can recommend suitable locations for emotional recovery such as campus parks, gyms, and study rooms according to generalized preferences and stress categories rather than personalized identifiers. Overall, this function supports student well-being by providing context-aware guidance while maintaining privacy protection.

## 4 Conclusion

Leveraging advanced digital technologies such as digital twins to address widespread mental health challenges, particularly anxiety-related stress among college students, has become an important research topic in public health. This study develops a campus digital twin prototype to visualize and manage anxiety and stress among college students, using Texas A&M University as a prototype deployment context to illustrate its potential feasibility for supporting campus mental health management. The proposed framework consists of three layers: data acquisition and management, modeling and simulation, and user applications. A web-based visualization dashboard prototype is developed for campus administrators, mental health professionals, and students. The system supports four primary functions: monitoring anxiety-related stress trends; campus stress heatmap visualization with spatiotemporal assessment and intervention; campus mental health facility management and spatial layout planning; and low-stress path navigation with restorative space recommendations. The proposed proof-of-concept DT prototype can support the monitoring of campus student mental stress conditions, improve the student restoration experience, and help reduce anxiety-related stress. In addition, it can enhance decision-making for campus mental health administrators and inform targeted public mental health intervention strategies in campus settings.

However, several limitations remain. First, this study is a proof-of-concept prototype at the design stage. We built this visualization system using synthetic data rather than real individual health data, which will need to be validated in practice during real-world deployment. Second, the system is still in the conceptual design phase, and user requirements and evaluations have yet to be incorporated into the prototype design. Based on user needs and feedback regarding the user experience, further updates will be required after the system is implemented. Third, the modeling and simulation layer is designed for rule-based scenario assignment rather than dynamic simulation. Modeling and simulation at finer temporal or spatial resolutions would require substantially greater computational power and incur higher costs. Finally, the collection and integration of individual-level mental health and location-related data raise important concerns regarding privacy protection, data security, and ethical governance.

Our future work will concentrate on using real-world health and environmental data to deploy and validate this digital twin framework and prototype. Evaluations involving multiple user groups, such as campus students, mental health professionals, and administrators, are necessary to understand their needs. In addition, privacy-preserving data aggregation, anonymization, and responsible data use will be essential in future implementations. Despite these remaining limitations, the proposed digital twin framework offers a scalable reference for advancing campus mental health research, while highlighting the importance of balancing technological innovation with data ethics and student privacy.

## Data Availability

The raw data supporting the conclusions of this article will be made available by the authors, without undue reservation.
